# Humans and Mice Express Similar Olfactory Preferences

**DOI:** 10.1371/journal.pone.0004209

**Published:** 2009-01-16

**Authors:** Nathalie Mandairon, Johan Poncelet, Moustafa Bensafi, Anne Didier

**Affiliations:** Université Lyon 1, CNRS, UMR5020, Neurosciences Sensorielles, Comportement, Cognition, Lyon, France; The Rockefeller University, United States of America

## Abstract

In humans, the pleasantness of odors is a major contributor to social relationships and food intake. Smells evoke attraction and repulsion responses, reflecting the hedonic value of the odorant. While olfactory preferences are known to be strongly modulated by experience and learning, it has been recently suggested that, in humans, the pleasantness of odors may be partly explained by the physicochemical properties of the odorant molecules themselves. If odor hedonic value is indeed predetermined by odorant structure, then it could be hypothesized that other species will show similar odor preferences to humans. Combining behavioral and psychophysical approaches, we here show that odorants rated as pleasant by humans were also those which, behaviorally, mice investigated longer and human subjects sniffed longer, thereby revealing for the first time a component of olfactory hedonic perception conserved across species. Consistent with this, we further show that odor pleasantness rating in humans and investigation time in mice were both correlated with the physicochemical properties of the molecules, suggesting that olfactory preferences are indeed partly engraved in the physicochemical structure of the odorant. That odor preferences are shared between mammal species and are guided by physicochemical features of odorant stimuli strengthens the view that odor preference is partially predetermined. These findings open up new perspectives for the study of the neural mechanisms of hedonic perception.

## Introduction

Olfaction is of great importance to mammals' survival, influencing a variety of social activities, including recognition, mate selection, fear responses to predator odors, and food intake [1]–[3]. Of the various aspects of olfactory perception, pleasantness is particularly fundamental, and dominates odor perception [4]. Most odors we encounter induce attraction or repulsion behavior. However, it is unclear what it is that makes a given component pleasant or unpleasant. While, in the visual and auditory modalities, perception can be predicted from the physical properties of the stimuli, the rules that govern the relationship between perception and chemical structure in olfaction are largely unknown, making it difficult to predict the perceptual properties of novel odorants. In a recent study, Khan and collaborators built a mathematical model to predict the hedonic valence of molecules in humans on the basis of their physicochemical properties. While it is well established that odor hedonic perception is strongly influenced by experience and learning [5], [6] and that its representations are characterized by a high level of plasticity [7]–[9], these authors suggest that it nevertheless remains partially dependent on the odorants' physicochemical properties [10].

This, if true, suggests a predetermination of odor preferences [10], and it might consequently be hypothesized that other species may show similar odor preferences to humans. The present study found that the same odorants were similarly attractive to mice and humans, revealing for the first time a component of olfactory preference conserved across the two species. Consistent with this, behavioral responses to the odorants (hedonic rating and sniffing in humans; investigation time in mice) were found to correlate with the physicochemical properties of the molecules, suggesting that olfactory preferences are indeed partially engraved in the structure of the odorant molecule. Our data support the view that odor preferences are partially predetermined, in contrast to the more common view of them as predominantly shaped by experience.

## Results

Our hypothesis was that humans and mice exhibit similar preferences towards the same odorants. To test it, we first assessed odor preference in humans (through odor pleasantness ratings) and in mice (through investigation time) and then investigated the relationship between these odor preferences and odorant structures.

Odorant selection was based on a recent study by Khan et al. [10]. Using principal component analysis (PCA, a multivariate statistical method), these authors generated two odor spaces:

1. A perceptual space generated from a matrix of 144 odorants and 146 verbal labels describing perceptual properties of odors (see Dravnieks [11]). Given the complexity of interpreting such a multidimensional matrix, PCA was applied to reduce this multidimensional space to a small number of principal components (PCs). The PCs are ordered so that each successive PC has the maximal possible variance, the first PC explaining the most variance of the original data set.
2. A physicochemical space generated from a matrix of 1565 odorants and 1513 physicochemical descriptors. Here again, PCA reduced this multidimensional space to a small number of PCs.

In the present study, the odorants used in Experiment 1 and in Experiment 2 were respectively from Khan's perceptual and physicochemical space.

### Mice and humans express similar odor preferences

#### Experiment in mice and humans using odorants selected according to their perceptual pleasantness (Experiment 1)

Odor preference was recorded using the same odorants in both species. In mice, odorant investigation time, a behavioral measure of the mouse's interest in and attraction for the odorant, was used as an index of odor preference: when smelling an attractive odor, mice spend more time investigating the odorant source than when encountering a less attractive odor [12], [13]. Odorants were presented to mice using a computer-assisted hole-board, and odorant investigation time (nose poking into the hole) was automatically recorded using electronic sensors [14]. Experiments in humans consisted in sniffing odorized vials and rating compound pleasantness, intensity and familiarity on a 9-point scale (“not at all” (1) to “extremely” (9) pleasant, intense or familiar).

**Figure 1:**
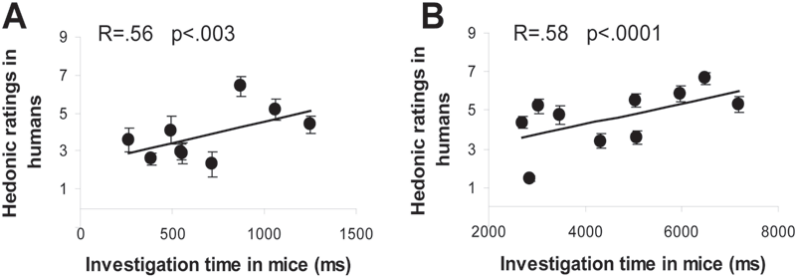
Mice and humans express similar odor preferences. A. In experiment 1, a significant and positive correlation was found between odor investigation time in mice and odor hedonic rating in humans. B. In experiment 2, using different odorants, a significant positive correlation was also observed between odor investigation time in mice and odor hedonic rating in humans.

In the first experiment, nine odorants were selected from the perceptual space of Khan et al. [10]. Multiple regression analysis (F[3,89] = 4.111, p<.001) revealed that the odorants investigated longest by mice were those rated most pleasant by humans (t(89) = 2.232, p<.003) (Fig. 1A). Mouse investigation time was also compared to other aspects of human olfactory perception represented by intensity and familiarity ratings (see Materials and Methods): no correlation was found, either with intensity (t(89) = 1.048, p>.05) or familiarity rating (t(89) = .340, p>.05). Of the different parameters of human olfactory perception measured here (hedonic, intensity and familiarity ratings), mouse investigation time thus correlated only with human olfactory pleasantness.

In brief, humans and mice showed similar preferences towards the nine odorants tested.

#### Experiment in mice and humans using odorants selected according to their physicochemical properties (Experiment 2)

To further investigate similarity in odor preference, the above experiment was replicated using ten different odorants, this time selected from the physicochemical rather than the perceptual space of Khan's study [10]. Again, a significant correlation between odor investigation time in mice and odor hedonic response in humans was observed (F[1,149] = 31.190, p<.0001) (Fig. 1B).

Pooling data from both experiments confirmed that odor investigation time in mice correlated positively with human hedonic ratings (F[1,239] = 41.709, p<.0001).

Importantly, in order to compare homologous behaviors in humans and mice, sniffing time, which reflects odor pleasantness [15]–[17], was recorded in humans, and found to correlate with investigation time in mice (F[1,239] = 15.535, p<.0001).

In brief, odor preferences correlated in mice and humans, whether perceptual or physicochemical criteria were used to select the odorants.

### Mouse and human odor preferences correlate with odorant structure

**Figure 2:**
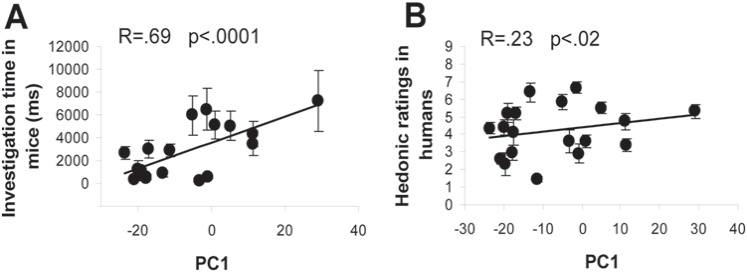
Mouse and human odor preferences are driven by odorant structure. Physicochemical PC1 correlated positively with investigation time in mice (A) and with hedonic rating in humans (B), indicating that hedonic behavior in both species may be driven in part by the physicochemical properties of the molecules.

A possible link between odor preference in humans or mice and odorant structure was explored using the first principal component (PC1, which explains the most variance) of the physicochemical space in Khan et al.'s study [10]. PC1 was found to correlate positively with both investigation time in mice (F[1,289] = 22.940, p<.0001) (Fig. 2A) and hedonic rating in humans (F[1,239] = 6.186, p<.02) (Fig. 2B). These results clearly link the physicochemical properties of odorants with their attractiveness in the two different species.

### Two groups of odorants with different physicochemical properties evoke distinct behaviors in mice and humans

**Figure 3:**
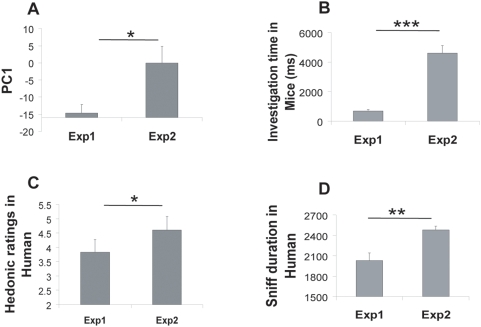
Two groups of odorants with different physicochemical properties evoke distinct behaviors in mice and humans. A. Physicochemical PC1 significantly differed for the two sets of odorants. B. Mice investigated odorants longer in experiment 2 than in experiment 1 C. Humans sniffed odorants longer in experiment 2 than in experiment 1 D. Humans preferred the odorants of experiment 2 to those of experiment 1. (t-test, *: p<.05, **: p<.001, ***: p<.0001)

Strengthening the above results, we observed differences in PC1 values between the first and the second experiment: the average value of PC1 across odorants in experiment 2 was significantly greater than that in experiment 1 (t-test, p<.05, Fig. 3A). As a positive PC1 value corresponds to increased investigation time in mice and increased odor pleasantness in humans (see Fig. 2), this difference may explain the distinct behaviors observed in response to the two sets of odorants used in experiments 1 and 2 (for both humans and mice): mice investigated longer and humans preferred and sniffed odorants longer in experiment 2 than in experiment 1 (t-test, p<.05, Fig. 3B–D). Taken together, these results support the view that odor preference is driven at least in part by the physicochemical properties of the molecules, accounting for the similar olfactory preferences found in both humans and mice.

## Discussion

Mammals possess an excellent ability to detect and discriminate odorants. They also exhibit odor preferences that seem to be crucial for survival [13], [18], [19] and represent a central component of the quality of human experience [20]. The present study examined whether odor preferences were similar between humans and mice, and might thus include a predetermined component.

A first result of interest was the positive correlation in odor preferences between mice and humans. Odor preferences result from complex physiological and motivational states characterized by experience-dependent plasticity in both animals [21], [22] and humans [4]. In particular, the hedonic representation of smells is not fixed and may be modified by learning and experience in both animals and humans [23]–[25]. For example, a smell may acquire a novel hedonic valence through an associative learning procedure [5], [26]. Moreover, it is well known in humans that odor pleasantness is modulated with repeated exposure to the same stimulus: i.e., without any apparent mediation of environmental stimuli [27]. Although hedonic representations are plastic, as seen above, the present demonstration that two different species exhibited similar preferences for the same odorants provides evidence for odor representations conserved across mice and humans, which emerge independently of life experience. These findings are in line with reports that human newborns exhibit olfactory preferences as shown by behavioral and physiological responses to chemical stimuli [28], and are able to exhibit behavioral markers of disgust in response to unpleasant odors [29]. Such predisposition in odor preference may be underlain by genetically programmed neural circuits, as has been suggested in the olfactory systems of mammals [13], *Drosophila melanogaster* [30] and *Caenorhabditis elegans* [31].

Secondly, the present investigation further showed that olfactory preferences in humans and mice are linked to the physicochemical structure of odorants. The data suggest that physicochemical properties are prominent factors in orchestrating the activation pattern of the predetermined neural code for odor preference. It may be asked why odorant structure should correlate more strongly with odor preference in mice than with hedonic perception in humans (F-values of 22.940 in mice, vs 6.186 in humans). One explanation may be that mouse preferences are very weakly colored by the environment (the laboratory mice were raised in an olfactory poor environment) whereas odor pleasantness in adult humans is likely to be more affected by learning and experience. Nevertheless, despite the strong influence of experience, the physicochemical properties of odorants still played a prominent role in determining odor preferences, strengthening Khan et al.'s model [10]. In other words, even if pleasantness is the result of culture, life experience and learning, the present interspecies comparison shows that there is an initial part of the percept which is innate and engraved in the odorant structure.

In conclusion, our phylogenetic heritage includes systems enabling the attribution of a positive or negative value, driving attraction to or avoidance of odorants. This suggests that, upstream of the hedonic plasticity occurring throughout life, we are endowed with a partly predetermined neural basis for these odor hedonic representations–even if the odorant has no biological significance, such as predator, conspecific or spoiled food odors [13], [29], [32]. Perception of the hedonic aspect of odorants is thus a complex process which involves both innate and learned components. Taken as a whole, these results substantially affect our view of olfactory hedonic perception and open up new avenues for the understanding of its neural mechanisms. They also suggest that odor exploration behavior in mice may be used to predict human olfactory preferences.

## Materials and Methods

### Experiments in mice

Adult (8-week old) male C57Bl6/J mice (Charles River Laboratories, L'Arbresle, France) were tested under procedures in accordance with the European Community Council Directive of 11/24^th^/1986 (86/609/EEC) and the French Ethics Committee.

Upon arrival in the lab, mice were housed in groups of five in standard laboratory cages and were kept on a 12 hr light/dark cycle (constant temperature), with food and water *ad libitum*. Experiments were conducted in the afternoon (2–5 pm) on a specially designed computer-assisted one-hole-board apparatus (40×40 cm; central hole 3 cm diameter, 4.5 cm deep), with capacitive sensors to detect automatically the beginning of each trial (when the mouse was placed in the starting area facing the hole) and monitor the duration of nose-poking into the hole. A polypropylene swab impregnated with 60 µL of odorant (1 Pa) was placed at the bottom of the hole, under a grid and covered with bedding [14]. The bedding was replaced after each trial. One odorant was presented per day (random order for each animal). Each trial lasted 2 min. Duration of nose-poking into the hole was used as a measure of odor preference. Ten mice were tested in the first experiment and twenty in the second.

### Experiments in humans

Respectively ten (mean age, 21.1 yr+/−1.07) and twenty human subjects (mean age, 21.85 yr+/−3.37) recruited from the University of Lyon (France) participated in experiments 1 and 2. Olfactory and/or neurological disease was the exclusion criterion. The study was conducted in accordance with the Declaration of Helsinki.

Testing was performed in an experimental room designed specifically for olfactory experiments. Odorants were presented in 15 ml flasks (opening diameter: 1.7 cm; height: 5.8 cm; filled to 5 ml) and were absorbed on a scentless polypropylene fabric (3×7 cm; 3 M, Valley, NE, USA) to optimize evaporation and air/oil partitioning.

After providing written informed consent to the procedure, which was approved by the “LyonSud-Est2” ethics committee, subjects were taken to the test room, where they sniffed each vial in random order and rated compound pleasantness on a 9-point scale (from 1: “not at all pleasant” to 9: “extremely pleasant”). Subjects rated compound intensity and familiarity (in experiment 1) on similar scales. The instructions given to the subjects were as follows: “You are going to smell several odors one after the other. Your task will be to sniff each vial and then to decide how intense, pleasant or familiar the smell was. To give your estimates, you will rate each odorant on a scale from 1 (not at all intense, familiar or pleasant) to 9 (very intense, familiar or pleasant).” Once the instructions had been read, the experimental session started. Odorants were presented every 45 sec.

Physiological data were acquired using a PROCOMP+ system (Thought Technology, Montreal, Canada; sampling rate, 32 Hz). Sniff duration was measured using an airflow sensor (AWM720, Honeywell, France) connected to nasal cannulae positioned in both nostrils [33] throughout the experimental sessions.

### Odorants

The nine odorants used in the first experiment and the ten used in second were diluted in mineral oil so as to achieve an approximate gas-phase partial pressure of 1 Pa (Table 1). Briefly, vapor pressures of pure odorants were estimated using ACD Chem-Sketch software (Advanced Chemistry Development, Toronto, Ontario, Canada) and variously diluted in mineral oil to concentrations theoretically emitting the same vapor-phase partial pressure for each odorant.

**Table 1:** Odors and their percentage (vol/vol) dilutions (1 Pa).

Experiment 1		Experiment 2	
acetophenone	0.56	hexanol3	0.07
amyl acetate	0.03	heptanol1	0.91
diphenyl oxide	13.55	thioglycolicAcid	0.32
ethyl butyrate	0.01	carvone-l	2.36
eugenol	13.12	geraniol	21.25
guaiacol	2.08	1-Decanol	33.73
heptanal	0.07	benzyl acetate	1.46
hexanoic acid	3.63	ionone βlowconc	30.60
phenyl ethanol	2.65	dodecanal	27.74
		santalol	14139.92

### Data analysis

Statistical analysis used SYSTAT software (SSI, Richmond, CA). In Experiment 1, “odor investigation time” in mice on the one hand and “odor pleasantness, intensity and familiarity” in humans on the other hand were compared by multiple regression analysis. In Experiment 2, “odor investigation time” in mice and “odor pleasantness” in humans were compared by simple regression analysis. Human sniff duration was compared to mouse investigation time using a similar analysis. Finally, the relation between “odorant PC1” and “investigation time” in mice (or “odor pleasantness” in humans) was also assessed by simple regression analysis.
